# A Systematic Review on Existing Measures for the Subjective Assessment of Rehabilitation and Assistive Robot Devices

**DOI:** 10.1155/2016/1048964

**Published:** 2016-05-04

**Authors:** Yiannis Koumpouros

**Affiliations:** Technological Educational Institute of Athens, Department of Informatics, Agiou Spyridonos, Aigaleo, 12243 Athens, Greece

## Abstract

The objective of the current study is to identify and classify outcome measures currently used for the assessment of rehabilitation or assistive robot devices. We conducted a systematic review of the literature using PubMed, MEDLINE, CIRRIE, and Scopus databases for studies that assessed rehabilitation or assistive robot devices from 1980 through January 2016. In all, 31 articles met all inclusion criteria. Tailor-made questionnaires were the most commonly used tool at 66.7%, while the great majority (93.9%) of the studies used nonvalidated instruments. The study reveals the absence of a standard scale which makes it difficult to compare the results from different researchers. There is a great need, therefore, for a valid and reliable instrument to be available for use by the intended end users for the subjective assessment of robot devices. The study concludes by identifying two scales that have been validated in general assistive technology devices and could support the scope of subjective assessment in rehabilitation or assistive robots (however, with limited coverage) and a new one called PYTHEIA, recently published. The latter intends to close the gap and help researchers and developers to evaluate, assess, and produce products that satisfy the real needs of the end users.

## 1. Introduction

The aging of society along with the lack of caregivers forces innovations to help people in their daily lives. Nowadays, robots come in many forms and can be used in many ways to help people with disabilities. Although much research has been done in the field resulting in several prototypes, few assistive robots exist in common use today. The high costs and the uncertainty regarding the benefits gained are major barriers to their widespread adoption. It is therefore crucial to follow a more multidisciplinary approach during the design phase. For example, an engineering design with only healthy subjects many times leads to a system not appropriate for the target population of persons with a disability. Engineers, therapists, physiatrists, and ergonomics experts as well as the end users (people with disabilities) should be a part of the design team from the beginning of any such effort. To this end, the design team should be able to measure the satisfaction of the end users at any stage of the development phase.

Measuring user satisfaction helps to measure the overall quality of a product or service. Tracking user satisfaction during the development phase can help developers and researchers make sure that the changes they are making improve the product/service for users. In customer relationship management, user (or customer) satisfaction is a measure of the degree to which a product or service meets the user's expectations. Consumer satisfaction is a central concept in many domains (business, research, etc.) and has held a central position in marketing since the 1950s until today, with an increasing interest and importance. The realization of this importance has led to a proliferation of research on consumer satisfaction [1–4]. According to [5] we can distinguish two different types of user satisfaction: the process-oriented approach (equal to the difference between expected satisfaction and achieved satisfaction) and the outcome-oriented approach (as an attribute extracted from a product or service after its consumption). The evaluation of any technology device requires the objective and subjective assessment of the product/service. An objective assessment is one that needs no professional judgment to give a score correctly, while subjective assessment yields many possible answers of varying quality and requires professional judgment to give a score [6]. Subjective assessment records the facts presented by the end user that show his/her perception, understanding, and interpretation of what is happening and therefore measures his/her satisfaction. The next step is to quantify them appropriately.

The evolution of Information and Communication Technologies (ICT) [7] along with nanotechnology and other sciences (e.g., medicine and behavioral science) presents a unique potential for innovative technology products and services that help people in their daily lives. The application of such innovations in healthcare has already driven in products that some years ago belonged to the scientific imagination sphere. For example, robotic technology has provided the opportunity to benefit the lives of people with disabilities (i.e., as a manipulator mounted on a desk or wheelchair or mobile base or body worn; as a mobility or communication assistant; etc.). Many commercial products are available nowadays for mobility and manipulation for people with physical disabilities (e.g., the Tek RMD [8] and the Manus robotic arm [9]), for telepresence purposes, and so forth [10], while others are being developed at research institutions worldwide. To this end, researchers are struggling to collect end users' sentiments towards the technologies developed in order to match the products with the real needs of the end customers.

Rehabilitation robotics is a special branch of robotics which focuses on machines that can be used to help people recover from severe physical trauma or assist them in activities of daily living. Rehabilitation robotics has applications in all areas of physical therapy, presenting a wide range of advancements in robotic prosthesis and other domains. Another important sector is that of assistive robotics, which tries to combine and integrate several technologies in order to meet the needs of people with various disabilities.

Focusing on the healthcare sector, quality of care and patient satisfaction are major issues [11]. Thus, the assessment of any service or device from the perspective of the patient is crucial. The current study is concerned with the question of whether any reliable and valid instruments have been developed to assess assistive or rehabilitation robot devices from the user's perspective. To the best of our knowledge, no such research has previously been carried out or published.

## 2. Materials and Methods

**Figure 1:**
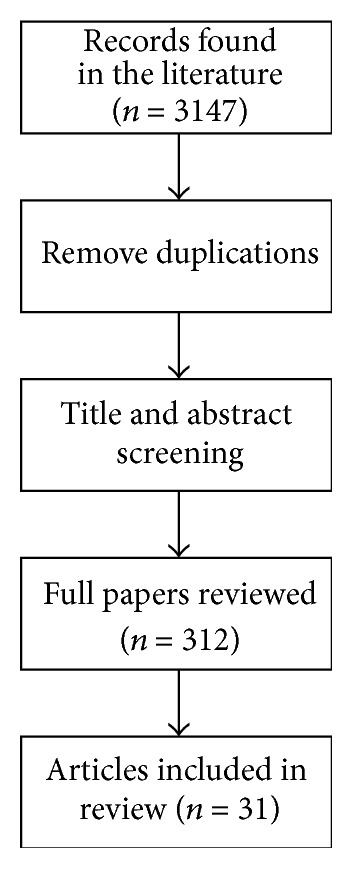
Workflow of the selection process for subjective assessment of rehabilitation and assistive robot devices.

A search of peer-reviewed published literature was conducted in January 2016 for articles related to the subjective assessment of assistive or rehabilitation robot devices. This was performed through the Internet via MEDLINE, PubMed, Scopus, and CIRRIE. The keywords used (employing Boolean phrases) in the searches included the following:* satisfaction*,* assessment*,* assess*,* user*,* subjective*,* robot*,* robotic*,* assistive*,* rehabilitation*,* psychometric*,* test*,* scale*,* metrics*,* evaluation*,* usability*,* acceptability*, and* acceptance*. Articles published after 1980 were considered for further studying. We included only studies published in English and research from peer-reviewed journals. The resulting publications were examined in a first step for potential inclusion based on their title and abstract, while we excluded any duplication. In a second step, after reviewing the abstract and title, the remaining publications underwent a full text review. We also manually searched the references lists of these articles for additional relevant sources. The whole process is depicted in Figure 1.

The initial query returned 3147 results. After the initial screening (reviewing the titles and abstracts and removing duplicates), the number was reduced to 312 articles. The eligibility criteria for inclusion were as follows: (i) studies involve assistive or rehabilitation robot devices (e.g., socially assistive robots, robot controlled wheelchairs, and exoskeleton devices), (ii) at least one outcome measure was used in the study, (iii) the study should include formative, process, or summative evaluation that assesses the rehabilitation or assistive robot device, (iv) the study was a peer-reviewed article (published in a journal or conference), and (v) articles are written in the English language. Finally, all articles were evaluated in terms of the clarity of the evidence presented. The second phase included a full text review of the selected articles. The main factors for excluding many articles included the following: (i) article was not written in English, (ii) the study provided a descriptive summary of the evaluation without giving more details (e.g., questionnaire/scale used), (iii) the number of participants in the study was very limited (<3 participants) or undefined, (iv) articles/studies were in the following subject areas: biochemistry, genetics and molecular biology, mathematics, chemical engineering, social sciences, materials science, physics and astronomy, psychology, arts and humanities, immunology and microbiology, agricultural and biological sciences, decision sciences, dentistry, energy, environmental science, business management and accounting, chemistry, earth and planetary science, pharmacology-toxicology and pharmaceutics, and undefined, (v) articles were in books and notes, and (vi) they assessed the technology used through quantitative data only (objective evaluation). For data extraction and analysis purposes we followed the Cochrane research methodology [12].

The processing and organization of the articles was made using the MS Excel and EndNote X6.0.2 programs.

## 3. Results

Even though the initial query returned a large number of articles (*n* = 3147), only 312 (9.914% of 3147) underwent a full text review. Finally, 31 articles (9.935% of 312) met the inclusion criteria and were included in the review.  Table 1 summarizes the subjective measures used in these studies.

A wide range of rehabilitation and assistive robot devices are reviewed in Table 1 (e.g., wheelchairs, exoskeleton devices, socially assistive robots, therapeutic systems, telepresence systems, and haptic technologies). The majority of the research presented was conducted in the United States of America (15.2%) and Italy (12.1%), followed by Canada (9.1%), Netherlands (9.1%), Japan (9.1%), France (9.1%), Spain (9.1%), the United Kingdom (6.1%), Germany (6.1%), South Korea (3%), Portugal (3%), Israel (3%), and Austria (3%).

**Figure 2:**
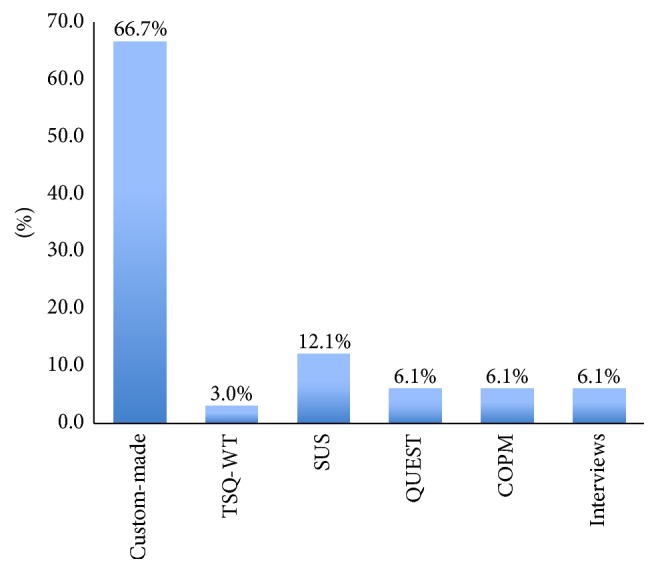
Subjective measure used in the studies reviewed (%).

In the majority of the studies there were a limited number of patients participating either due to research/pilot testing or due to difficulty finding the right participants fitting in the study. Only one study managed to have 83 patients [13]. Twenty-two out of the thirty-one studies used patients to evaluate the device, while three recruited college or university students, two recruited healthcare professionals, and the remaining four used healthy subjects. Additionally, two of the studies used healthy subjects as control groups.  Figure 2 presents the distribution of the subjective measures used in the studies.

The last column of Table 1 presents the number of items (questions) used in each study under the subjective measure used. The question mark “?” in Table 1, when used, means that there is no evidence (at least to the authors' knowledge) that the specific subjective measure/scale is valid or reliable. The authors decided to use this instead of marking the specific measure as not valid or not reliable because the articles report the scale as valid. However, after searching the literature, we found no evidence on the validity and reliability of the scale in the language used in the specific study; so, we decided to present it as being in question in terms of its validity and reliability in the language used. Moreover, in the last column of the table there may appear two values separated by a comma. This means that the study used two different subjective measures and each value in the column represents the number of items (questions) used for each one of them.

## 4. Discussion

The review of the literature provided a thorough survey of evaluations for rehabilitation or assistive robot devices concerning the satisfaction and the subjective assessment of the end users. The review began with a great number of articles returned (*n* = 3147) but was ultimately narrowed to 312 of which 31 were finally included in the review. The majority of the articles failed to meet the inclusion criteria presented because most of the studies evaluated the devices by using only objective measures (e.g., Fugl-Meyer scale [14], modified Ashworth scale [15], Barthel index [16], and NASA-TLX [16]) and other clinical measurements relevant to the scope and the population of the study.

It is really impressive that the great majority (66.7%) of the research included in the review used custom-made subjective measures to evaluate the satisfaction of the end users and assess the rehabilitation or assistive robot device used subjectively [17–38]. However, this percentage does not reveal the real problem faced. Someone should also consider the following facts:

- Two (2) studies [39, 40] used semistructured interviews due to the lack of any valid and reliable subjective measurement instrument.
- Two (2) studies [13, 41] used the Canadian Occupational Performance Measure (COPM) [42] which is a semi-interview that enables an open dialogue between the end user and the therapist on issues of importance to the patient. It is designed to identify occupational performance problems faced by the patient. During the interview, the therapist tries to identify daily occupations of importance that the patient wants to do, needs to do, or is expected to do but is unable to accomplish. Other domains are also covered, like self-care, productivity, or leisure. Due to the nature of COPM, as described above, it cannot be adopted in all kinds of purpose rehabilitation or assistive devices.
- Four (4) studies [30, 43–45] used the System Usability Scale (SUS) [46] which is a “quick and dirty” ten-item scale for administering after usability tests. However, this scale measures only basic issues of the device and does not take into account other very important issues, like the adaptability of the device, the feeling of safety, the social perception of the user when using the device, the individual dimensions of the device, if it fits well in the environment of the end user, and so forth. So, this scale can capture only very basic issues of the subjective assessment needed for robot-based devices.
- One (1) study [43] utilized the Telehealthcare Satisfaction Questionnaire-Wearable Technology (TSQ-WT) [47]. There has not yet been published (at least to the authors' knowledge) any article in any language related to the validity and reliability of this questionnaire. Thus, it cannot be considered a reliable and valid instrument for the subjective assessment of a technology device.
- Two (2) studies [48, 49] utilized the Quebec User Evaluation of Satisfaction with Assistive Technology (QUEST 2.0) scale [50]. According to Holz et al., QUEST 2.0 is a standardized satisfaction assessment tool designed for assistive technologies [51]. Moreover, it has been tested for its validity and reliability in several applications and languages [52, 53]. Although this scale is used with assistive devices, del-Ama et al. [48] used only 7 of the 12 questions incorporated in the original questionnaire in their study.

According to the previous analysis, it is apparent that finally 93.9% of the reviewed studies used neither valid nor reliable instruments to assess the robot devices. Only 6.1% of them used a validated measure (the QUEST scale), which can assess only a subset of the desired aspects (as described in the next paragraphs).

In the early years of many technical fields, the research community often utilizes a wide range of metrics that are not comparable due to a bias towards application specific measures. The primary difficulty in defining common metrics is the incredibly diverse range of human-robot or robot assisted applications. Thus, although metrics from other fields (HCI, human factors, etc.) can be applied to satisfy specific needs, identifying metrics that can accommodate the entire application space may not be feasible [54].

Attempts to categorize both objective and subjective metrics have been made. According to the USUS Evaluation Framework for Human-Robot Interaction [55] the factors* usability*,* social acceptance*,* user experience*, and* societal impact* are considered the main categories of evaluation factors. Each category is divided into specific metrics, either objectively or subjectively measured:

- *Usability*. Effectiveness, efficiency, learnability, flexibility, robustness, and utility.
- *Social Acceptance*. Performance expectancy, effort expectancy, attitude towards using technology, self-efficacy, forms of grouping, attachment, and reciprocity.
- *User Experience*. Embodiment, emotion, human-oriented perception, feeling of security, and coexperience with robots.
- *Societal Impact*. All effects of the introduction of robotic agents' consequences for the social life of a specific community (taking into account cultural differences) in terms of quality of life, working conditions and employment, and education.

Among these, the authors propose that the following could be tested using end-user questionnaires, which means that they could be considered subjective:

- *Utility*. It refers to how an interface can be used to reach a certain goal or to perform a certain task. The more the tasks the interface is designed to perform, the more the utility it has.
- *Performance Expectancy*. It is the degree to which an individual believes that using the system will help him or her to attain gains in performance.
- *Effort Expectancy*. It indicates to which extent the user perceives that a system will be easy to use.
- *Attitude towards Using Technology*. It is the sum of all positive or negative feelings and attitudes about solving working tasks supported by a humanoid robot.
- *Self-Efficacy*. It relates to a person's perception of their ability to reach a goal.
- *Attachment*. It is an affection-tie that one person forms between themselves and another person or object—a tie that binds them together in space and endures over time.
- *Reciprocity*. It is the positive or negative response of individuals towards the actions of others.
- *Embodiment*. It describes the relationship between a system and its environment and can be measured by investigating the different perturbatory channels like morphology, which has impact on social expectations.
- *Emotion*. An emotion is an essential part in social interaction; it has to be incorporated in the assessment and design of robots.
- *Feeling of Security*. It is important to investigate how to design human-robot interactions in a way that humans experience them to be safe.
- *Coexperience*. Coexperience describes experiences with objects regarding how individuals develop their personal experience based on social interaction with others.
- *Societal Impact*. Societal impact describes all effects the introduction of robotic agents results in for the social life of a specific community—taking into account cultural differences—in terms of quality of life, working conditions, employment, and education.

The above categorization is the most full and detailed, including aspects that are rarely taken into account when it comes to evaluating a robotic assistant. Most researchers, however, when evaluating an assistive device/technology tend to use questionnaires that give information on the aforementioned fields. However, as Bartneck et al. mention, due to their naivety and the amount of work necessary to create a valid questionnaire, developers of robots have a tendency to quickly cook up their own questionnaires [56]. This conduct results in two main problems: firstly, the validity and reliability of these questionnaires have often not been evaluated and, secondly, the absence of standard questionnaires makes it difficult to compare the results from different researchers [56]. The findings of our review study support this conclusion. It seems that choosing tailored questionnaires is the rule in robotics assessment. However, the existing variety of questionnaires that could be useful for the assessment of rehabilitation or assistive robot devices is narrow, for the reasons described above.

As derived from the current review, QUEST 2.0 may be one questionnaire that can be used in the examined field. However, a strategy that would target maximum coverage of the subjective measures spectrum would require the combined use of two (or more) questionnaires, since QUEST 2.0 covers only some subjective aspects (mainly: feeling of security, perceived effectiveness, and ease of use). We should therefore look into the literature for other valid and reliable scales that are used in different sectors and may be applicable to the examined domain. Other valuated and relevantly common used questionnaires we found in the bibliography were the Assistive Technology Device Predisposition Assessment- (ATDPA-) Device Form [57, 58] and the Psychosocial Impact of Assistive Devices Scale (PIADS) [59]. The ATDPA-Device Form is more relevant in context than the PIADS, targeting the evaluation of overall user experience with assistive technology, while PIADS only emphasizes the psychosocial impact of assistive devices, without targeting the evaluation of the actual experience of interacting with a robot device, but rather the impact that this interaction has on quality of life (QoL). Other questionnaires such as the USE-IT [60] questionnaire were ruled out from the very beginning, since they were not well valuated or not widely used from researchers in the bibliography. It seems therefore that a combination of the QUEST 2.0 questionnaire and the Assistive Technology Device Predisposition Assessment- (ATDPA-) Device Form covers most of the desirable user-experience aspects, with ensured validity and reliability. However, no scales have been identified yet in the literature that could be adopted well and measure the individual functionalities of rehabilitation or assistive robot devices. To this end, the authors developed and are currently examining a new scale called PYTHEIA in order to fill the identified gap. The first results are very satisfactory in terms of their validity and reliability [61]. More specifically, according to the results from the exploratory factor analysis (EFA) with varimax rotation performed, the PYTHEIA instrument presents a three-factor model (the “Independent Functionalities” factor, the “Fit to Use,” and the “Ease of Use” factors). The overall Cronbach *α* of PYTHEIA was found to be 0.793, indicating sufficient consistency. The ICC was excellent (ICC = 0.992, *p* = 0.000), indicating that the PYTHEIA total scores were highly consistent between initial assessment and reassessment. The paired-samples *t*-test between the two instances of administration indicated no statistically significant systematic bias (*p* = 0.059). Pearson's *r* correlation coefficient indicated stability of participants' responses over time (Pearson's *r* = 0.984, *p* = 0.000). Examination of item convergent validity showed that all item intercorrelations for all item pairings were strong or excellent. Pearson's *r* ranged from 0.946 to 0.996 for the first factor “Independent Functionalities,” from 0.465 to 0.724 for the second “Fit to Use,” and from 0.354 to 0.732 for the third factor “Ease of Use.” This provides evidence that all subscales' items are related to the same construct.

## 5. Conclusions

In this paper we conducted a systematic review of the literature in order to identify existing scales for the subjective assessment of rehabilitation and assistive robot devices. We found that most of the studies are utilizing either custom-made questionnaires or interviews that are neither valid nor reliable instruments to represent the subjective opinion and perception of the end users. There is therefore a great gap in the subjective assessment of rehabilitation or assistive robot devices. The absence of standard scales/questionnaires for the subjective assessment of robot-based devices makes it difficult to design products that meet exactly the needs of the intended end users, to further improve prototypes, or to compare the results from different researchers. Based on the findings of the review, in order to further improve the subjective assessment of rehabilitation and assistive robot devices it is necessary for each study to (i) select as subjects the appropriate target group based on clear and valid inclusion criteria, (ii) involve a sufficient number of representative subjects, (iii) analyse statistically the collected data, and (iv) select an established methodology in order to enable comparison between results of different studies.

## Figures and Tables

**Table 1:** Summary of review results.

Robot device used	Region/country	Author/organization (year of publication), publisher [ref. number]	Number of patients/end users	Type of end users	Location of the survey	Subjective measure used	Language of scale used	Valid/reliable scale	Number of scale's items
A sensory system and upper limb biomechanical model combined with a graphical interface	Ontario, Canada	Abdullah et al. (2011), J. Neuroeng. Rehabil. [17]	20	Patients	Inpatient Stroke Rehabilitation Unit	Unknown (developed by themselves)	English	—/—	2
Robotic exoskeleton arm	Italy	Ambrosini et al. (2014), Robotica [43]	14	9 patients + 5 healthy	Villa Beretta Rehabilitation Centre	TSQ-WT, SUS	Italian	?/?, ?/?	TSQ-WT (30), SUS (10)
Hand/wrist exoskeleton	United Kingdom, Netherlands, Italy	Amirabdollahian et al. (2014), Robotica [44]	12	Patients	Not defined	SUS	English	V/R	10
H2 robotic exoskeleton	Not defined	Bortole et al. (2015), J. Neuroeng. Rehabil. [18]	3	Patients	Not defined	Unknown (developed by themselves)	English	—/—	1
Haptic human-robot partnered stepping	Atlanta, GA, USA	Chen et al. (2015), PLoS ONE [19]	10	Healthy	Healthcare Robotics Lab	Unknown (developed by themselves)	English	—/—	14
Direct physical interface for nursing assistant robots	Atlanta, GA, USA	Chen and Kemp (2010), HRI 2010 [20]	18	Healthy (nurses)	Healthcare Robotics Lab	Unknown (developed by themselves)	English	—/—	11, 10
Robot suit HAL (Hybrid Assistive Limb)	Tokyo, Japan	Chihara et al. (2016), Neurol. Med. Chir. [39]	15	Patients	Kyoto University Hospital	Interviews			
Robot “El-E”	Atlanta, GA, USA	Choi et al. (2008), ASSETS '08 [21]	8	Patients	Healthcare Robotics Lab	Unknown (developed by themselves)	English	—/—	8
SAM robotic aid system (a mobile Neobotix base equipped with a semiautomatic vision interface and a Manus robotic arm)	France	Coignard et al. (2013), Annals of Phys. and Rehab. Med. [22]	29 + 34	29 patients + 34 healthy (control group)	Hopale Foundation in Berck-sur-Mer and the Kerpape Rehabilitation Centre in Ploemeur	Unknown (developed by themselves)	French	—/—	9 (technical aspects), 7 (acceptability and usage)
Hybrid FES-robot (exoskeleton)	Spain	del-Ama et al. (2014), J. Neuroeng. Rehabil. [48]	4	Patients	Not defined	QUEST	English	V/R	7 from 12
Wheelchair mounted robotic assisted transfer device	Pittsburgh, USA	Grindle et al. (2015), BioMed Res. Int. 2015 [23]	18	Patients	2011 National Veteran Wheelchair Games	Unknown (developed by themselves)	English	—/—	4, 7
iCat robot	Netherlands	Heerink et al. (2010), Int. J. Soc. Robot. [24]	30	Healthy	Not defined	Unknown (based on the UTAUT questionnaire)	English	—/—	41
ARM, HEXAR-KR40P	South Korea	Kim et al. (2014), Int. J. Precis. Eng. Man. [25]	80	Patients	Not defined	Unknown (developed by themselves)	English	—/—	1
A-gear: wearable dynamic arm support	Netherlands	Kooren et al. (2015), J. Neuroeng. Rehabil. [26]	4	3 patients + 1 healthy	Radboud UMC Outpatient Clinic	Unknown (developed by themselves)	Not defined	—/—	Not defined
Grasping robot	France	Laffont et al. (2009), Arch. Phys. Med. Rehabil. [27]	20 + 24	20 patients + 24 healthy (control group)	Four French departments of physical and rehabilitation medicine	Unknown (developed by themselves)	French	—/—	3
Haptic-robotic platform for upper limb	Canada	Lam et al. (2008), J. Neuroeng. Rehabil. [28]	8	Healthy (physical and occup. therapists)	Not defined	Unknown (developed by themselves)	English	—/—	9
Teleoperated robot system Telenoid R3	Japan	Liu et al. (2015), HRI 2015 [29]	20	Healthy (college students)	ATR Intelligent Robotics and Communication Labs, Kyoto	Unknown (developed by themselves)	Japanese	—/—	2
LEGO robot	Spain	Lopez-Samaniego et al. (2014), Bio-Med. Mater. Eng. [30]	9	Patients	Not defined	Unknown (developed by themselves), SUS	Spanish	—/—, ?/?	Not defined, SUS (10)
InMotion 2 robotic system	Italy	Mazzoleni et al. (2014), Comput. Methods Programs Biomed. [31]	34	Patients	Not defined	Unknown (developed by themselves)	Italian	—/—	7
Personal Transport Assistance Robot (PTAR)	Japan	Ozaki et al. (2013), Arch. Phys. Med. Rehabil. [32]	8	Patients	Fujita Health University	Unknown (developed by themselves)	Japanese	—/—	2
Rehabilitation robot	Canada	Pineau et al. (2010), Advances in Intelligent and Soft Computing [33]	7	Healthy (university students)	Not defined	Unknown (developed by themselves)	Not defined	—/—	Not defined
Amadeo robot	Italy	Sale et al. (2012), Stroke Res. Treat. [41]	7	Patients	Department of Neurorehabilitation, IRCCS San Raffaele Pisana	COPM			
Robot-enhanced repetitive treadmill therapy (ROBERT)	Germany	Schroeder et al. (2014), Dev. Med. Child Neurol. [13]	83	Patients	Not defined	COPM			
Robot companion (artificial health advisor)	Germany	von der Pütten et al. (2011), ICMI '11 [40]	6	Healthy	University of Duisburg-Essen	Semistructured interviews			
Personal Mobility and Manipulation Appliance (PerMMA)	USA	Wang et al. (2013), Med. Eng. Phys. [34]	15	Patients	Center for Assistive Technology, University of Pittsburgh	Unknown (developed by themselves)	English	—/—	12
Kompaï (indoor assistive robot)	France	Wu et al. (2014), Clin. Interv. Aging [35]	11	Patients	Living lab	Unknown (developed by themselves)	English	—/—	25
ASIBOT (portable robot to aid patients)	Spain	Jardón et al. (2011), Disabil. Rehabil. Assist. Technol. [49]	6	Patients	Not defined	QUEST	Spanish	V/R	12
Intelligent wheelchair	Portugal	Mónica Faria et al. (2013), Assist. Technol. [45]	46	Healthy (students)	School of Allied Health Sciences of Porto	SUS	Portuguese	?/?	10
Socially assistive robot (Nao)	Austria	Werner and Krainer (2013), ICSR 2013 [36]	14	Healthy	Senior Citizen Centre Schwechat	Unknown (developed by themselves)	German	—/—	Not defined
Reo Therapy System	Israel	Treger et al. (2008), Eur. J. Phys. Rehab. Med. [37]	10	Patients	Loewenstein Rehabilitation Centre	Unknown (developed by themselves)	Not defined	—/—	15
Robotic and electrical stimulation therapy	United Kingdom	Hughes et al. (2011), Disabil. Rehabil. Assist. Technol. [38]	5	Patients	Not defined	Unknown (developed by themselves)	English	—/—	Not defined

Note: TSQ-WT = Telehealthcare Satisfaction Questionnaire-Wearable Technology, SUS = System Usability Scale, QUEST = Quebec User Evaluation of Satisfaction with Assistive Technology, UTAUT = Unified Theory of Acceptance and Use of Technology, COPM = Canadian Occupational Performance Measure, ? = unknown value, — = not valid (if it appears in the first position of the column “Valid/reliable scale”)/not reliable (if it appears in the second position of the column “Valid/reliable scale”), V = valid scale, and R = reliable scale.
